# Influence of Nursing Intervention on Recurrent Vulvovaginal Candidiasis Patients Treated with ATP-infrared Bio-effect Technique

**Published:** 2018-10

**Authors:** Wenxiang LI

**Affiliations:** Dept. of Gynecology, Women & Children’s Health Care Hospital of Linyi, Linyi, China

**Keywords:** Nursing intervention, ATP-infrared bio-effect technique, Recurrent vulvovaginal candidiasis, Quality of life

## Abstract

**Background:**

We aimed to investigate the influence of nursing intervention on recurrent vulvovaginal candidiasis (RVVC) patients treated with adenosine triphosphate (ATP)-infrared bio-effect technique.

**Methods:**

Sixty eight RVVC patients of Women & Children’s Health Care Hospital of Linyi were randomly divided into intervention group (n=34) and control group (n=34) from to June, 2016 to June 2017. Patients in intervention group received the one-to-one intervention based on ATP-infrared bio-effect technique; the individualized nursing intervention program was developed. Patients in control group were treated with routine nursing for vaginitis and follow-up. The intervention effect was evaluated via clinical efficacy and MOS 36-item short-form health survey (SF-36) scale.

**Results:**

After the total course of treatment, there was significant difference in the cure rate between the two groups of patients (*P*<0.05). 1 month after all courses of treatment, the scores of physical functioning (PF), mental health (MH), vitality (V) and bodily pain (BP) in intervention group were higher than those in control group, displaying statistically significant differences (*P*=0.029; *P*=0.049; *P*=0.042; *P*=0.039, respectively). 6 months after the total course of treatment, the score of each dimension in intervention group was higher than that in control group during the same period (*P*<0.01).

**Conclusion:**

Nursing intervention can increase the cure rate, change the adverse cognition and improve the quality of life of RVVC patients treated with ATP-infrared bio-effect technique.

## Introduction

Recurrent vulvovaginal candidiasis (RVVC) refers to the attack of mycologically-confirmed symptomatic VVC for 4 times or above within 1 year, whose incidence rate is about 5% (1). It is manifested as pruritus vulvae, increased vaginal secretions, soybean curb-like or curd-like secretions, burning pain, dyspareunia, dysuria and other VVC symptoms. Due to repeated attack or ineffective treatment, RVVC brings serious troubles to the patient’s mental health, sexual life and social functions, affecting daily life and family relationship and causing a heavy psychological burden to patients. RVVC has always been a difficulty and hot spot in VCC research, but the research on RVVC nursing has not been paid enough attention to.

At present, clinical treatment methods of RVVC include intensive therapy and consolidation therapy, in which the consolidation therapy is performed for 6 months after mycological cure via intensive therapy (2). However, there are some problems, such as longer treatment cycle, poor compliance of patients, unsatisfactory results and high recurrence rate.

The concept of holistic nursing development in modern nursing has been expanded to physical, psychological, family, social, spiritual and cultural levels, but the research on RVVC nursing has not been paid enough attention to. In this study, RVVC patients treated with adenosine triphosphate (ATP)-infrared bio-effect technique were examined, and the nursing intervention was performed during treatment according to the patient’s understanding of disease and psychological status, so as to observe the impact of nursing intervention on patients.

## Materials and Methods

### Objects of study

A total of 68 RVVC patients treated in the Gynecological Clinic of Women and Children’s Health Care Hospital of Linyi, China from June 2016 to June 2017 were enrolled, and randomly divided into intervention group (n=34) and control group (n=34). They were aged (35.25±10.12) years old on average, including 8 patients aged <30 years old and 60 patients aged >30 years old. In terms of educational level, there were 19 cases of junior college and above, 14 cases of senior high school, 18 cases of junior high school and 17 cases below junior high school. The course of disease was 1–3 years, and all patients were married or unmarried women with a history of sexual life.

The study was approved by the ethics committee of Women & Children’s Health Care Hospital of Linyi and informed consents were signed by the patients and/or guardians.

Inclusion criteria: 1) patients meeting diagnostic criteria of RVVC, with the chief complaints of genital and/or vaginal itching, burning sensation, dyspareunia and increased vaginal secretions, 2) patients with vulvovaginal congestion, edema or rupture, soybean curb-like or curd-like secretions covered on vaginal mucosa and cervical surface, and red and swollen mucosa or mucosal erosion exposed after being wiped, 3) blastospore and pseudohypha could be seen in secretions via 10% potassium hydroxide solution wet film method or Gram staining microscopy, 4) patients with the course of disease for more than 1 year, and the disease recurrence for at least 4 times within 1 year, 5) patients who did not take other antibacterial drugs within half a month before treatment and received no systemic anti-infective and anti-fungal treatment during the test, and 6) patients who could adhere to the therapeutic regimen and follow-up on time.

Exclusion criteria: 1) pregnant woman, 2) patients with severe heart, liver, kidney or blood disease, 3) patients who were allergic to drugs used in this study, 4) patients who breached the therapeutic regimen and still had sexual life during treatment, or 5) patients who were unwilling to cooperate in the follow-up.

### Methods

Patients in intervention group were treated with the nurse-led intervention measures based on routine nursing. The nurse-led intervention measures refer to the well-defined comprehensive intervention measures different from routine nursing, in which gynecological nurses serve as intervention project managers and resource coordinators to provide consultation, interview, reminder and health education for RVVC patients, so as to improve the RVVC treatment adherence. Patients in control group were treated with routine nursing, namely the traditional RVVC nursing service developed and implemented in our department. All patients in control group and intervention group received the same nursing services in other aspects, except the nurse-led intervention measures.

Intervention group: (a) ATP treatment was divided into 3 steps: 1) Rinsing: The special micro-ecological anti-bacterial inhibitor I and 50% glucose were prepared into the macromolecular colloid penetrating fluid. The vagina was first rinsed with penetrating fluid (if the entire vagina was filled with secretions, it was rinsed with bottled water for injection before being rinsed with penetrating fluid), and then rinsed again with micro-ecological anti-bacterial inhibitor I. During rinsing, the vaginal circular folds should be paid attention to, and the angle of speculum was adjusted constantly, so that the hidden secretions could be fully removed. Then the vagina was thoroughly wiped dry with large cotton swabs. 2) Irradiation: After rinsing, the inner-middle vagina, anterior and posterior vaginal walls and side wall were irradiated by using the ATP-IR bio-effect therapy apparatus (Beijing ATP Medical Technology Co., Ltd.) for a total of 5 min. The power was set as 30–40 based on the treatment grade during irradiation, and the probe was adjusted. The irradiation distance was adjusted at any time according to the patient’s feeling. 3) Medicine applying: After treatment, 500,000 units of nystatin tablets and 0.2 g metronidazole tablets were placed into the vagina once a day for intensive treatment for 7 d as 1 course of treatment, followed by consolidation therapy for 2 courses of treatment according to the menstrual cycle. (b) Intervention program: The one-to-one intervention was adopted, and the individualized nursing intervention program was developed based on the patient’s symptom characteristics and the experience of professionals. The intervention measures were made by a trained nurse at the right time. The specific intervention measures are as follows:

(I) Questionnaire
  All selected patients filled out the self-designed questionnaire, and the personal files were created. The content of questionnaire included name, age, occupation, hobby, education level, dietary habit, health habit, frequency of sexual activity, condition of sexual partner, contraceptive measures, previous history, history of menstruation, history of pregnancy and birth and medication, dressing habits, and the application of protection pad in non-menstrual period. The patient’s cognition degree of VVC and psychological status were evaluated.
(II) Assessment: Subjective indexes included the patient’s cognition of disease, subjective symptoms, psychological status and processes of medical care, treatment and medication. Objective indexes included the examination results of vaginal secretions, degree of vulva and vaginal mucosal inflammatory response, absence or presence of swelling and ulceration, color, quantity, character and odor of vaginal secretions.
(III) Inquiry: The patient’s educational level, personal hygiene, vaginal cleaning habits, frequent vaginal lavage, frequency of abortion, oral administration of contraceptives, placement of intrauterine device, application of broad-spectrum antibiotics, immunosuppressants and estrogen therapy recently, absence or presence of hot spring and hip bath habits, examination results of blood glucose, dressing habits, replacement frequency of beddings, house orientation, etc., were understood.
(IV) Propaganda and education: According to the evaluation results and personal data, the individualized health education was provided in straightaway language to explain the related knowledge to VVC and RVVC, including the etiology, pathogenic characteristics and predisposing factors of VVC, route of transmission, treatment time, knowledge of medication and precautions, so as to strengthen the patient’s understanding of disease and improve the compliance with medication. 1) Elimination of etiological factors: Patients who received the broad-spectrum antibiotics, immunosuppressants and estrogen therapy recently should consult a professional doctor to decide whether to withdraw the drug according to the specific conditions, and patients with high blood glucose should pay attention to controlling the blood glucose. 2) Personal hygiene: Patients were asked to clean the underpants, boil them in boiled water and then dry them, and they were also asked to replace the bedding and sofa cushions. The washing machine was cleaned by professionals. 3) Diet: The poor eating habits were changed; spicy and stimulating food, especially red pepper, should be avoided, and the intake of sugar and chocolate should be reduced. Excessive intake of sugar-rich foods, such as glucose, arabinose and ribose, will lead to VVC more easily, because they are the carbon sources of Candida albican. Lactose can be converted to ribose and arabinose, so the excessive intake of it will also result in VVC (3). 4) Vaginal washing: The frequent excessive vaginal washing should be avoided. The frequent washing is closely related to the upper genital tract infection and pelvic infection, and the long-term vaginal washing can lead to cervical cancer, pelvic inflammation, premature birth, infertility, RVVC or ectopic pregnancy (4). 5) Dressing: It is appropriate to wear the loose, comfortable and breathable underpants, and avoid using sanitary pads during non-menstrual period. Otherwise, the local humidity and temperature in the perineum will be increased, thus creating a favorable environment for the reproduction of Candida albican (pH 4–5, 37°C). Combined with the poor ventilation in vulva and the mucosal friction injury, the vaginal environment may be changed, inducing RVVC. However, wearing loose and breathable cotton underpants can remove moisture and effectively prevent the repeated attack of VVC (5). 6) Personal hobbies: It was forbidden to take a bath in hot spring and swim before the disease was cured. 7) Education of medical compliance: Patients were informed of the importance of standardized treatment, and it was forbidden to take drugs repeatedly. 8) Guidance of sexual life: The frequency of sexual life should be reduced. Candida albican, as the conditioned pathogen, can also be parasitic in the oral cavity, intestines and other parts of the human body, besides vagina. In addition to cross infection, it can also be directly transmitted through sexual intercourse. Some studies (6) have shown that the occurrence of RVVC is related to the frequent sexual activity recently. After sexual intercourse, seminal fluid can induce Candida albican to form pathogenic mycelium, and sperm may block the immune response of lymphocytes to Candida albican. Moreover, sperm may contribute to the colonization of Candida albican with cross infection through sexual life, leading to the repeated attack of VVC in females (7). Therefore, reducing sexual intercourse is helpful to reduce the incidence of VVC. In principle, conventional treatment is not necessary for sexual partners, but about 15% men will suffer from balanitis after sexual contact with VVC patients (1), so male partners should also receive the examination and treatment of Candida albican to prevent repeated infection in females. It is needed for both partners to clean the vulva before and after sexual intercourse and avoid oral sex.
  “Three-no” principles should be followed during treatment: no sexual intercourse, no washing and no medication. In other words, the sexual activity and vaginal washing should be avoided during treatment, and other vaginal drugs should be withdrawn. The vaginitis health education brochure was distributed.
(V) Individualized nursing: RVVC patients suffer from great physical and psychological pain due to repeated attack of disease, and they are too embarrassing to describe the disease due to private part, ultimately delaying treatment. After the release of two-child policy, patients with demand for pregnancy, especially those older patients, are eager to have a second child, leading to high anxiety and depression. The long-term impact on sexual life even affects the husband-wife relation. A 43-year-old rural patient was eager to have a second child. But she suffered from the disease and sought treatment everywhere. She got up at 3 am every day and went to hospital by bus with long-term medication, causing physical fatigue and mental depression on the verge of collapse. During treatment, examination results of her vaginal secretions were analyzed through the patient’s description and objective observation. Doctors gave her systematic guidance and communicated with her at the right time and place using the plain language, so as to change her adverse cognition. All courses of treatment were completed, and the disease did not relapse after follow-up for 6 months. The patient came to the hospital to express her thanks with a renewed mental outlook. During operation, the doctor should listen carefully to the patient’s description, communicate with the patient to understand his psychological conditions and personality traits, talk gently in view of the individual psychological characteristics and expectations, exchange with the patient according to his educational level and understanding ability using the plain and acceptable language, provide correct solutions to different questions and comfort the patient’s mood, so that the patient can really feel the concern for her and realize that RVVC can be completely cured, thus reducing the psychological pressure on them and actively cooperating with treatment under good psychological conditions.
(VI) Follow-up: Patients were followed up at 1 month, 3 months and 6 months after all courses of treatment.
  Control group: After routine vulva cleaning, 500,000 units of nystatin tablets and 0.2 g metro-nidazole tablets were placed into the vagina once a day before sleep every night for intensive treatment for 7 d as 1 course of treatment. Then consolidation therapy was performed for 2 courses of treatment according to the menstrual cycle. Moreover, RVVC routine nursing was also performed; the vaginitis health education brochure was distributed, and patients were followed up in the same frequency in intervention group.
  Efficacy evaluation:
  (I) Determination of clinical efficacy
    According to the patient’s clinical symptoms and signs and relevant laboratory examination results, the clinical efficacy was determined based on “*Diagnostic Basis of Clinical Disease: Criteria of Cure and Improvement*” (8):
    Efficacy index (N) = {(total score before treatment - total score after treatment)/total score before treatment} × 100%.
    1) Cured: All clinical symptoms and signs disappear, and Candida albican is negative in laboratory examination. The score of clinical symptoms and signs declines, N≥95%.
    2) Remarkably effective: Clinical symptoms and signs are relieved significantly, and Candida albican is negative in laboratory examination. The score of clinical symptoms and signs declines, 95%>N≥70%.
    3) Effective: Clinical symptoms and signs are relieved slightly, and Candida albican is positive in laboratory examination. The score of clinical symptoms and signs declines, 70%>N≥30%.
    4) Ineffective: Clinical symptoms and signs have no change or are aggravated, and Candida albican is positive in laboratory examination. The score of clinical symptoms and signs has no change or does not decline significantly, N<30%.
  (II) MOS 36-item short-form health survey (SF-36) scale (9, 10)

The scale includes 8 dimensions and 36 items, including 1) physical functioning (PF), 2) role-physical (RP), 3) mental health (MH), 4) role-emotional (RE), 5) social functioning (SF), 6) vitality (V), 7) bodily pain (BP) and 8) general health (GH). The Cronbach’s α coefficient is 0.78–0.94. The score ranged from 0 to 100 points, and the higher the score is, the higher the quality of life will be. At the time of treatment, the questionnaire and relevant baseline data were filled out by trained nurses, patients were followed up on time, and results were recorded.

### Statistical methods

Statistical Product and Service Solutions (SPSS) 17.0 software (Chinese version) was used for data processing in this study. *t* test was used for measurement data; chi-square test was used for enumeration data, and Ridit analysis was used for ranked data. Repeated measures analysis of variance, *t* test or Mann-Whitney test was used for the comparison of measurement data between the two groups, such as baseline data and quality of life at 1 month, 3 months and 6 months after all courses of treatment. *P*<0.05 suggested that the difference was statistically significant.

## Results

### Comparisons of general data of patients

There were no statistically significant differences in age, course of disease, symptoms and other general data between the two groups, and data were comparable.

### Comparisons of RVVC treatment results between the two groups before and after intervention

After all courses of treatment, the total effective rate was not significantly different between the two groups of patients, and there was a statistically significant difference in the cure rate (*P*<0.05) (Table 1).

**Table 1:** Comparison of total clinical efficacy between the two groups of patients after treatment [n (%)]

***Group***	***Case (n)***	***Cured***	***Remarkably effective***	***Effective***	***Ineffective***	***Total effective rate (%)***
Intervention group	34	24 (70.59)	6 (17.65)	2 (5.88)	2 (5.88)	94.12
Control group	34	12 (35.29)	7 (20.59)	8 (23.53)	7 (20.59)	79.41

### Comparison of quality of life between the two groups of RVVC patients before and after intervention

According to results of repeated measures analysis of variance, there were statistically significant differences in the time effect, intergroup effect and interaction of each dimension of quality of life of RVVC patients (*P=*0*.*042*,* 0.037,0.026 respectively).

The comparisons of indexes between the two groups at each measurement point revealed that the scores of PF, BP, V and MH of patients in intervention group were higher than those in control group at the time of intensive therapy, showing statistically significant differences (*P*<0.01). In the follow-up at 3 months, only the GH score had no statistically significant difference. In the follow-up at 6 months, the score of each dimension in intervention group was higher than that in control group during the same period, displaying statistically significant differences (*P*<0.01) (Table 2).

**Table 2:** Comparison of quality of life between the two groups of patients before and after intervention (points, x̄±s)

***Item***	***Group***	***Before intervention***	***1 month after treatment***	***3 months after treatment***	***6 months after treatment***	**F *time***	**F *intergroup***	**F *interactio***
PF	Intervention group	84.36±12.35	83.67±11.32^*^	87.21±12.98^*^	90.36±14.23^**^	1018.019^*^	110.047^*^	97.872^*^
	Control group	84.89±12.98	81.45±11.69	81.35±12.86	82.45±14.16			
RP	Intervention group	68.82±24.92	70.78±26.23	79.02±25.23^*^	83.45±26.13^**^	334.674^*^	78.876^*^	101.096^*^
	Control group	68.73±25.08	69.66±26. 98	71.34±25.46	72.34±26.56			
MH	Intervention group	65.21±14.78	66.13± 14.65^*^	68.64±15.72^*^	72.56±16.68^**^	868.192^**^	170.401^**^	89.102^**^
	Control group	66.12±15.02	60.14±14.04	61.23±15.42	62.45 ±16.11			
RE	Intervention group	65.12±29.56	72.13±32.34	76.21±33.17^*^	78.25± 32.13^**^	198.365^*^	91.432^*^	139.689^*^
	Control group	65.23±29.34	69.21±33.05	68.13 ±33.28	67.62±32.28			
SF	Intervention group	76.78±19.36	70.46±21.12	76.19± 19.48^*^	82.19± 22.07^**^	242.864^*^	123.682^*^	88.268^*^
	Control group	77.23±20.02	70.78± 21.45	71.82±20.03	73.08±22.15			
V	Intervention group	63.56± 12.35	67.37±15.64^*^	68.12±15.55^**^	71.38±15.19^**^	340.684^**^	127.092^**^	129.681^**^
	Control group	63.28±12.61	61.82±15.28	59.92±15.49	61.49±15.22			
BP	Intervention group	80.75±16.33	78.19±16.59^*^	80.68±16.84^*^	86.03±16.24^**^	680.873 ^**^	79.892^*^	78.121^*^
	Control group	80.63±16.42	76.36±16.81	78.83±16.25	79.15±16.37			
GH	Intervention group	61.38±15.72	60. 76±15.69	60.29±15.67	67.54±16.28^**^	192.089	85.212	77.827
	Control group	61.90±16.11	60.03±15.77	60.18±15.98	61.05±16.56			

* *P<*0.05,

** *P*<0.01

## Discussion

Drug therapy is always a preferred choice for RVVC patients in clinic, but the role of nurses during the treatment process is ignored. Nursing intervention plays a decisive role in the psychological, family and social impacts caused by the disease.

The ATP-infrared bio-effect therapeutic apparatus used in this study is based on the theory of infrared bio-effect (11–14). In the natural broad spectrum, there is a wavelength with the same energy as that of intracellular ATP, so it is known as ATP light. ATP light can act on the amide bond on the protein directly, and supply energy to cells instead of ATP, so that cells can obtain and use energy immediately.

Basic theoretical research has demonstrated that the synthesis and utilization of energy are indispensable for all vital activities, and the energy supply for biological activity mainly comes from the energy released in the hydrolysis of ATP molecules on the protein (15–19).

If the protein energy supply is insufficient, and cells cannot develop normally for some reason, the protein in cells can be activated by using a specific wavelength (the energy at this wavelength is equivalent to that released in ATP hydrolysis) in the infrared spectrum to supply energy to epithelial cells, so biological tissues will return to normal (20).

During operation, the vagina must be washed thoroughly. In case there are many secretions filling the entire vagina, the vagina can be rinsed with water for injection first, and then washed by using the macromolecular colloid penetrating fluid prepared by special micro-ecological regulator and glucose. The operation should be performed gently, so as to avoid increased bleeding due to further stimulus against ulcer. During irradiation, the position of probe and vaginal dilator should be constantly adjusted, so as to ensure irradiation from different angles and increase the irradiation area, thus improving the curative effect.

Currently, the therapeutic regimen in the *Regulation of Diagnosis and Treatment of VVC (Revised)* in 2012 is adopted in the treatment of RVVC. For patients taking anti-fungal drugs for a long time, monitoring of hepatic-renal function should also be paid attention to. During the long-term pathogenic therapy, the anti-fungal drugs have obvious side effects and produce drug resistance easily, which disturb the micro-ecological balance of vagina with poor compliance of patients.

It is clinically found that the recurrence mechanism of most RVVC patients remains unclear, and the recurrence is often accompanied with the decline in autoimmune mechanism and changes in micro-ecological environment of vagina. The host plays an important role in RVVC. RVVC patients are always treated with drug therapy. The improvement of vaginal micro-ecosystem has been paid attention to in recent years, but the role of nurses during treatment is ignored.

ATP-infrared bio-effect treatment is noninvasive, which can avoid drug resistance due to excessive treatment and long-term medication, so it is easily accepted by patients. The micro-ecological regulator used in it is a kind of special micro-ecological regulator, which can improve the micro-ecological environment of vagina (21, 22). The micro-ecological anti-bacterial regulator I and 50% glucose are prepared into the fluid at a concentration of 70% in the first treatment, and then the concentration is reduced to 50%, 50% and/or 30% based on local conditions of patients (23–25). The inflammatory tissue fluid in the affected site is replaced via osmosis, which, as the pretreatment before irradiation, helps cells in the affected site return to normal and improves the micro-ecological environment of vagina. Observing the patient’s condition is one of the nurse’s duties, and they must carefully observe the characteristics and changes in the patient’s vulva and vaginal lesions every time during operation, adjust the concentration of flushing fluid according to the specific conditions, and smear the regulator II to patients with severe pruritus vulvae using cotton swabs.

At present, the domestic research on the treatment of RVVC though ATP-infrared bio-effect technique still needs to be further discussed. The treatment steps include the preparation of flushing fluid, flushing and irradiation, during which nurses should seize such a favorable opportunity and take full advantage of this moment to understand the patient’s condition through effective communication, and provide individualized education and guidance. At this time, patients are often in anxiety and depression state, and it is urgent for them to understand the concept of holistic nursing development in modern nursing, which has been expanded to physical, psychological, family, social, spiritual and cultural levels. However, the research on RVVC nursing is not paid enough attention to. In the treatment of RVVC patients with ATP-infrared bio-effect technique, nurses can give full play to professional expertise, implement holistic nursing, pay attention to the unity and integrity of the human mind and body, and provide nursing intervention according to the patient’s understanding of disease and psychological state while improving the local micro-ecological environment of vagina, thereby curing the disease fundamentally, making the patient’s physical and mental health in the best state and improving the quality of life of patients via the harmonious unity of treatment and nursing.

## Conclusion

Nursing intervention has a significant effect on the treatment of recurrent vulvovaginal candidiasis by ATP-infrared biological effect technology. It can improve the cure rate of RVVC, reduce the mental pressure and improve the quality of life. It is an ideal choice for the patients of RVVC.

## Ethical considerations

Ethical issues (Including plagiarism, informed consent, misconduct, data fabrication and/or falsification, double publication and/or submission, redundancy, etc.) have been completely observed by the authors.
